# Targeting viperin improves diet-induced glucose intolerance but not adipose tissue inflammation

**DOI:** 10.18632/oncotarget.20724

**Published:** 2017-09-08

**Authors:** Zhengtang Qi, Jie Xia, Xiangli Xue, Jiatong Liu, Weina Liu, Shuzhe Ding

**Affiliations:** ^1^ The Key Laboratory of Adolescent Health Assessment and Exercise Intervention, Ministry of Education, East China Normal University, Shanghai 200241, China; ^2^ College of Physical Education and Health, East China Normal University, Shanghai 200241, China

**Keywords:** viperin, glucose intolerance, adipose tissue, inflammation, lipid metabolism

## Abstract

Viperin is an interferon-inducible antiviral protein, responsible for antiviral response to a variety of viral infections. Here, we show that silencing viperin by antisense oligonucleotides (ASO) protects against diet-induced glucose intolerance, and yet exacerbates adipose tissue inflammation. In high-fat diet-fed mice, viperin ASO improves glucose homeostasis, reduces plasma triglyceride concentrations and ameliorates diet-induced hepatic steatosis. Peripheral delivery of viperin by adeno-associated virus elevates fasting plasma glucose and insulin concentrations and reduces insulin-stimulated glucose uptake in skeletal muscle. Viperin overexpression reduces epinephrine- stimulated lipolysis in white adipose tissue, whereas viperin ASO increases expression of lipolytic genes. Targeting viperin by antisense oligonucleotides promotes reciprocal regulation of hepatic and adipose lipogenesis by reducing hepatic lipid content and increasing triacylglycerol content in adipose tissue. These findings reveal viperin as an important target to improve glucose metabolism, and suggest that suppressing antiviral potential may improve the metabolic adaptability to high-fat diet.

## INTRODUCTION

Type 2 diabetes and obesity are associated with chronic inflammation characterized by increased proinflammatory responses and macrophage infiltration into adipose tissues [1, 2]. Therefore, anti-inflammatory treatments have beneficial effects on glycemia and insulin resistance. However, the anti-inflammatory effects of antidiabetes drugs are inconsistent by reducing hyperglycemia [3]. This suggests chronic inflammation is not the real cause for impairing glucose homeostasis in diabetes. In contrast, proinflammatory response is essential for innate immunity against viral and bacterial infections. The proinflammatory response not only resists pathogens but also orchestrates tissue remodeling with metabolic homeostasis [4]. For example, adipose tissue-specific deficiency in proinflammatory potential resulted in reduced visceral fat, impaired intestinal barrier function, increased hepatic steatosis, and metabolic dysfunction [5]. Whether adipose tissue inflammation represents a consequence or a cause of insulin resistance remains an open question. In general, viruses induce chronic inflammation by activating proinflammatory signaling and promote the development of obesity [6]. However, adenovirus 36 (Ad36)-induced obesity in mice increases angiogenesis in adipose tissue and improves glycemic control, regardless of increased adiposity and inflammation [7]. Hepatitis C virus (HCV) infection is associated with obesity, diabetes, and nonalcoholic fatty liver disease and increases the risk of progression to hepatic fibrosis [8, 9]. HCV directly causes insulin resistance independent of the visceral adipose tissue area in non-obese and non-diabetic humans [10]. Moreover, eradicating HCV ameliorates insulin resistance without any accompanying change in abdominal fat depots [11]. These abnormal phenotypes suggest that antiviral response directly impairs insulin sensitivity in host cells and tissues, thereby impairing glucose homeostasis independent of obesity and adipose tissue inflammation.

Successful antiviral therapy can induce remission of type 2 diabetes. Recent case reports in humans suggest that antiviral treatment of HCV improved glycemic control in type 2 diabetes [12, 13]. Viperin (cig5) is an IFN-inducible antiviral protein that inhibits genome replication of a number of viruses. Viperin could be induced in a variety of cell types by interferon (IFN), DNA and RNA viral proteins, and polysaccharide [14]. Viperin localizes to lipid droplets via its N-terminal alpha-helix [15], by which viperin inhibits HCV replication by binding to HCV at the lipid-droplet interface [16]. In addition, human cytomegalovirus (HCMV) resulted in viperin relocalization from the endoplasmic reticulum to mitochondria, where viperin interacted with trifunctional enzyme subunit beta (mitochondrial), reduced fatty acid beta-oxidation and ATP generation, and increased lipogenesis. HCMV-induced viperin disrupts cellular metabolism to enhance infectious process [17, 18]. Targeting viperin to mitochondria in the absence of HCMV replicated all of these metabolic outcomes, indicating that viperin is an independent regulator of lipid metabolism [18]. Thus, viperin is a potential target for antiviral treatments of diabetes by linking antiviral activity to metabolic process.

We evaluated the expression pattern of viperin in several tissues of insulin-resistant mice, which indicated that adipose tissues express the lower levels of viperin. However, specific knockdown of viperin with antisense oligonucleotides (ASO) in HFD-fed mice ameliorated glucose tolerance and enhanced adipose tissue inflammation. Viperin overexpression elevated fasting plasma glucose and inhibited adipocyte lipolysis. Silencing viperin induced reciprocal regulation of hepatic and adipose lipid metabolism under the challenge of high-fat diet. These findings suggest a previously unknown function for viperin in linking glucose metabolism to antiviral activity.

## RESULTS

### Viperin expression is downregulated in adipose tissue of insulin resistant mice

**Figure 1:**
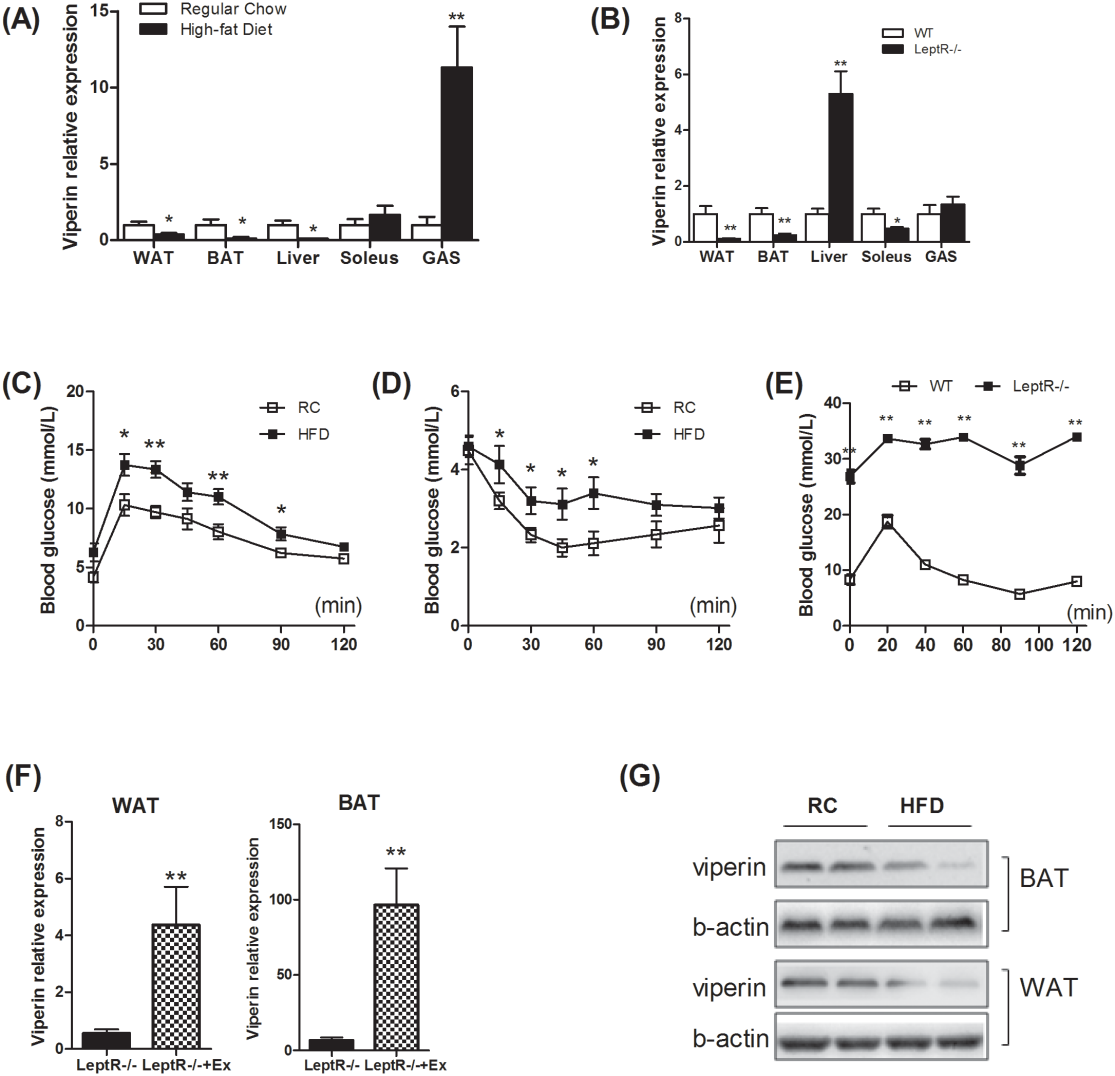
Insulin resistance reduced viperin expression in white and brown adipose tissues Mice were fed HFD for 3 months to induce insulin resistance. LeptR^-/-^ mice, leptin receptor- deficient db/db mice characterized initially by insulin resistance, were fed chow diet to determine viperin expression in different tissues. Relative mRNA expression of viperin was quantified in white adipose tissue (WAT), brown adipose tissue (BAT), liver, soleus and gastrocnemius (GAS) in HFD-fed mice **(A)** and LeptR^-/-^ mice **(B)**. Glycemic response to glucose tolerance test **(C)** and to insulin tolerance test **(D)** was examined in HFD-fed mice at the end of the study. **(E)** Glycemic response to glucose tolerance test was examined in LeptR^-/-^ mice at the end of the study. Relative mRNA expression of viperin in WAT/BAT was investigated in LeptR^-/-^ mice subjected to 6-wk swimming exercise **(F)**, which was performed in a water tank for 60 min/day. Relative mRNA expression is normalized to GAPDH in liver and muscle and to 18S in adipose tissue by 2^−ΔΔCt^ method. The data shown in Panels A–F are mean ± SE of 7–8 mice per group. ^*^P<0.05, ^**^P<0.01 versus control mice respectively. **(G)** Viperin expression in mice exposed to HFD was also investigated by western blot. Western blot shows that long-term exposure to HFD decreases the expression of viperin protein in WAT and BAT.

To understand gene expression profiling of viperin in obesity and insulin resistance, we first determined viperin expression in different tissues in HFD-fed mice and leptR^-/-^ mice (db/db mice). In the two mouse models of insulin resistance, viperin expression was consistently decreased in WAT and BAT (Figure 1A, 1B), whereas changes in liver and skeletal muscle were not consistent (Figure 1A, 1B). Glucose and insulin tolerance test indicated that these mouse models were insulin resistant and glucose homeostasis was impaired (Figure 1C-1E). We next examined the effects of 6-week regular exercise on viperin expression in leptR^-/-^ mice. Exercise increased viperin expression in WAT and BAT in leptR^-/-^ mice (Figure 1F). These changes may be associated with exercise effects on insulin resistance. In addition, viperin protein levels in WAT and BAT were both reduced in HFD-fed mice (Figure 1G). Of all these tissues, viperin expression in adipose tissue best relates to obesity and glucose intolerance in mice. Thus, we proposed that silencing viperin in adipose tissue may impair glucose homeostasis by reducing antiviral potential.

### Viperin knockdown protects against diet-induced glucose intolerance

**Figure 2:**
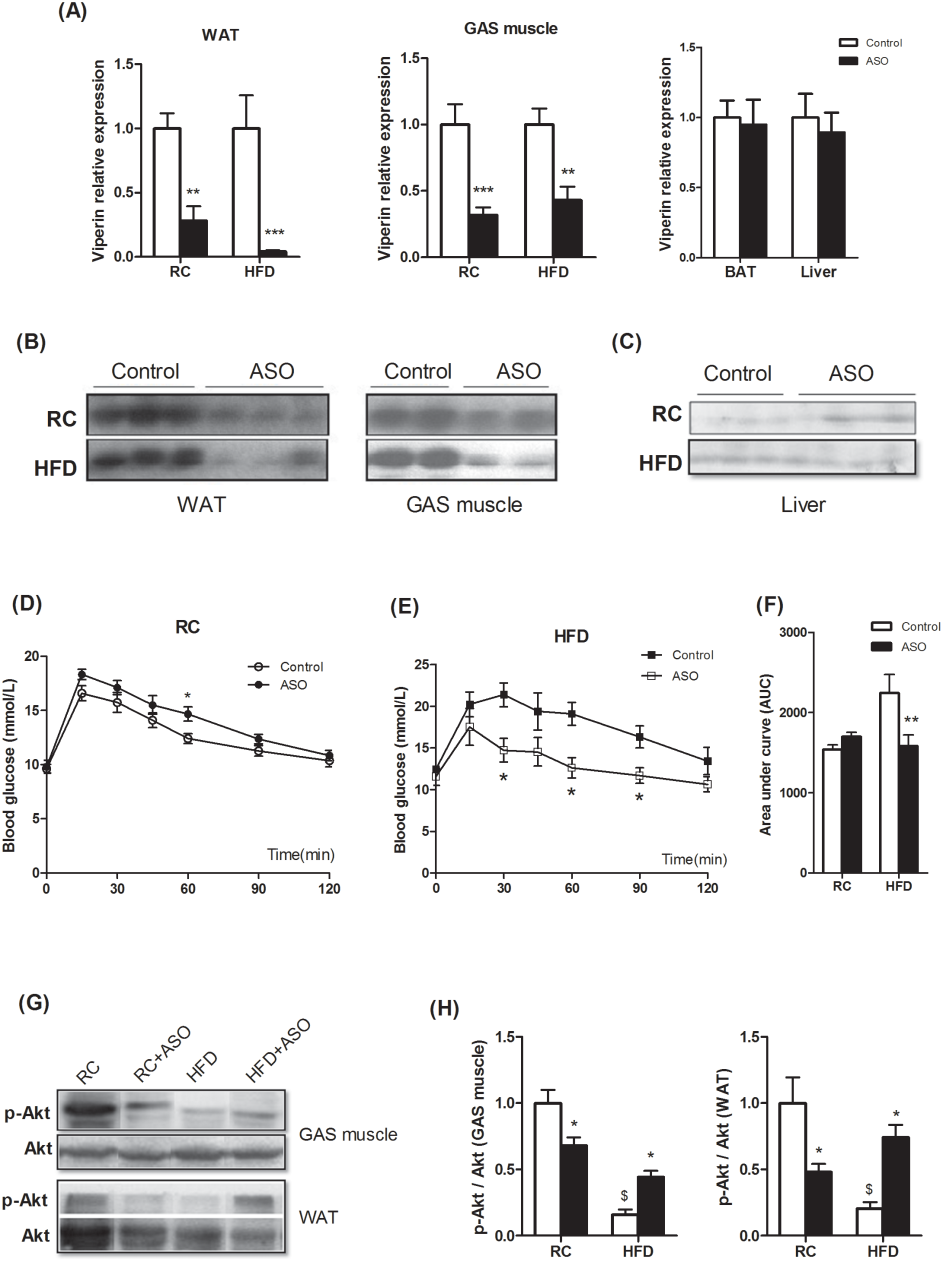
Viperin knockdown improved diet-induced glucose intolerance Mice received i.p injections of ASO against Viperin or a scrambled control ASO twice a week for 4 wks. Meanwhile, mice were fed HFD for 4 wks. Viperin ASO decreased viperin mRNA expression in WAT and skeletal muscle, but not in BAT and liver **(A)**. Values are normalized to 18S by 2^−ΔΔCt^ method. The data shown are mean ± SE of 5-6 mice per group. ^**^P<0.01, ^***^P<0.001 versus control mice respectively. Western blot shows that viperin ASO reduced viperin protein levels in WAT and skeletal muscle **(B)**, but the protein level of viperin in liver is too low to detect **(C)**. The data show that viperin ASO targeted WAT and skeletal muscle and silenced viperin expression. Panels D–F showed glycemic response to glucose tolerance test (IPGTT) in regular chow (RC)- and HFD-fed mice at the end of the study **(D, E)**. Area under curve in Figure 2D-2E was shown to evaluate glucose intolerance **(F)**. The data shown are mean ± SE of 5-6 mice per group. ^*^P<0.05, ^**^P<0.01 versus control mice respectively. **(G)** Phospho-Akt (Ser473) in WAT and GAS was investigated by western blot. Western blot shows that viperin ASO rescued HFD-induced inhibition of Akt phosphorylation. The blot shown is representative of 2 or 3 others showing the same pattern. **(H)** The quantitative analysis of western blot in Figure 2G. The data shown are mean ± SE of 3-4 mice. ^*^ P<0.05, versus control mice fed the same diet. ^$^ P<0.05, versus control mice fed RC.

To determine the extent to which viperin controls glucose homeostasis *in vivo*, we treated regular chow(RC)- and HFD-fed mice with viperin ASO twice a week for 4 weeks. Antisense oligonucleotides (ASOs) are well designed to bind to the target RNA and reduce expression of the target gene primarily in liver and adipose tissue [19]. Here, treating mice with viperin ASO reduced viperin mRNA/protein expression by 70-90% in WAT and 50-60% in skeletal muscle (Figure 2A, 2B). However, viperin mRNA expression was not decreased in BAT and liver (Figure 2A). The protein level of viperin was too low to detect in liver (Figure 2C), so liver did not show a significant reduction in viperin expression. This prompted us conclude that viperin ASO reduced expression of the target gene by targeting WAT and skeletal muscle, rather than BAT and liver.

In contrast to our hypothesis, viperin knockdown did not lead to a significant glucose intolerance in chow-fed mice (Figure 2D). Instead, viperin knockdown improved glucose intolerance (Figure 2E, 2F) and significantly reduced serum triglycerides in HFD-fed mice (Table 1). Additional assessments of fat/muscle weight, food intake, plasma glucose, FFA and cholesterol were performed after ASO treatment. Two-way ANOVA showed that sources of variation were associated with interactions between ASO and diets in most of metabolic parameters, such as body weight, liver weight, gastrocnemius weight, BAT weight, serum triglycerides and FFA (Table 1). Significant interaction suggested that effects of viperin ASO really depended on diet conditions. In addition, HFD reduced Akt phosphorylation in WAT and GAS muscle, whereas this reduction was partly rescued by viperin ASO in HFD-fed mice (Figure 2G, 2H). Together, viperin knockdown protects against diet-induced glucose intolerance and ameliorates hyperlipemia.

**Table 1:** Basal metabolic parameters in control and ASO mice fed regular chow and high-fat diets

	Regular chow		High-fat Diet		Two-way ANOVA		
	Control	ASO	Control	ASO	ASO	Diet	Int
Body and tissue weight							
Bodyweight(g)	21.9 ± 0.46	22.4 ± 0.47	24.7 ± 0.31	22.7 ± 0.58†		^**^	^*^
Heart(mg)	118.0 ± 4.98	124.0 ± 5.84	129.5 ± 1.03	121.4 ± 4.82			
Liver(mg)	867.2 ± 35.52	879.2 ± 34.69	1008.0 ± 14.02	876.8 ± 34.09†		^*^	^*^
WAT(mg)	477.3 ± 22.76	483.2 ± 48.74	476.0 ± 17.84	597.6 ± 31.44†			
Kidney(mg)	153.7 ± 6.79	153.6 ± 6.28	175.5 ± 3.41	157.8 ± 4.43		^*^	
Gastrocnemius(mg)	248.0 ± 12.09	225.6 ± 12.74	227.0 ± 13.77	259.0 ± 9.38			^*^
Quadriceps(mg)	273.2 ± 15.33	283.4 ± 9.08	298.8 ± 16.00	288.4 ± 12.37			
BAT(mg)	135.8 ± 6.41	134.6 ± 12.97	104.5 ± 5.74	99.2 ± 14.14		^**^	
WAT/bodyweight(mg/g)	21.8 ± 1.11	21.5 ± 1.90	19.3 ± 0.70	26.6 ± 1.86††	^*^		^*^
BAT/bodyweight(mg/g)	6.2 ± 0.24	6.0 ± 0.54	4.2 ± 0.25	4.4 ± 0.58		^***^	
Gastrocnemius/bodyweight(mg/g)	11.3 ± 0.40	10.1 ± 0.47	9.2 ± 0.61	11.5 ± 0.43††			^**^
Quadriceps/bodyweight(mg/g)	12.4 ± 0.59	12.7 ± 0.34	12.1 ± 0.68	12.7 ± 0.41			
Food intake (g/day)	3.5 ± 0.14	3.6 ± 0.19	3.9 ± 0.12	3.5 ± 0.11			
Plasma							
Glucose(mmol/L)	7.1 ± 0.17	6.2 ± 0.44	5.9 ± 0.42	5.9 ± 0.50			
Triglycerides(mmol/L)	1.8 ± 0.23	2.2 ± 0.21	7.8 ± 0.67	1.6 ± 0.23††	^***^	^***^	^***^
Free fatty acid(mmol/L)	1.3 ± 0.11	0.9 ± 0.09	0.6 ± 0.11	0.9 ± 0.12		^**^	^**^
HDLc(mmol/L)	3.3 ± 1.00	2.8 ± 0.90	3.6 ± 0.70	4.4 ± 0.89			
LDLc(mmol/L)	0.9 ± 0.16	0.7 ± 0.07	0.7 ± 0.30	0.8 ± 0.12			
Total cholesterol(mmol/L)	2.3 ± 0.28	2.5 ± 0.26	3.3 ± 0.13	3.0 ± 0.20		^**^	

Data are means ± SEM. Int, interaction. ^*^P<0.05, ^**^P<0.01, ^***^P<0.001 for the respective sources of variation (ASO, diet, or their interaction) using two-way ANOVA.^†^P<0.05, ^††^P<0.01 vs. controls on the same diet after a Bonferroni post hoc test (n = 5~6).

### Viperin overexpression increases fasting plasma glucose and reduces skeletal muscle glucose uptake

**Figure 3:**
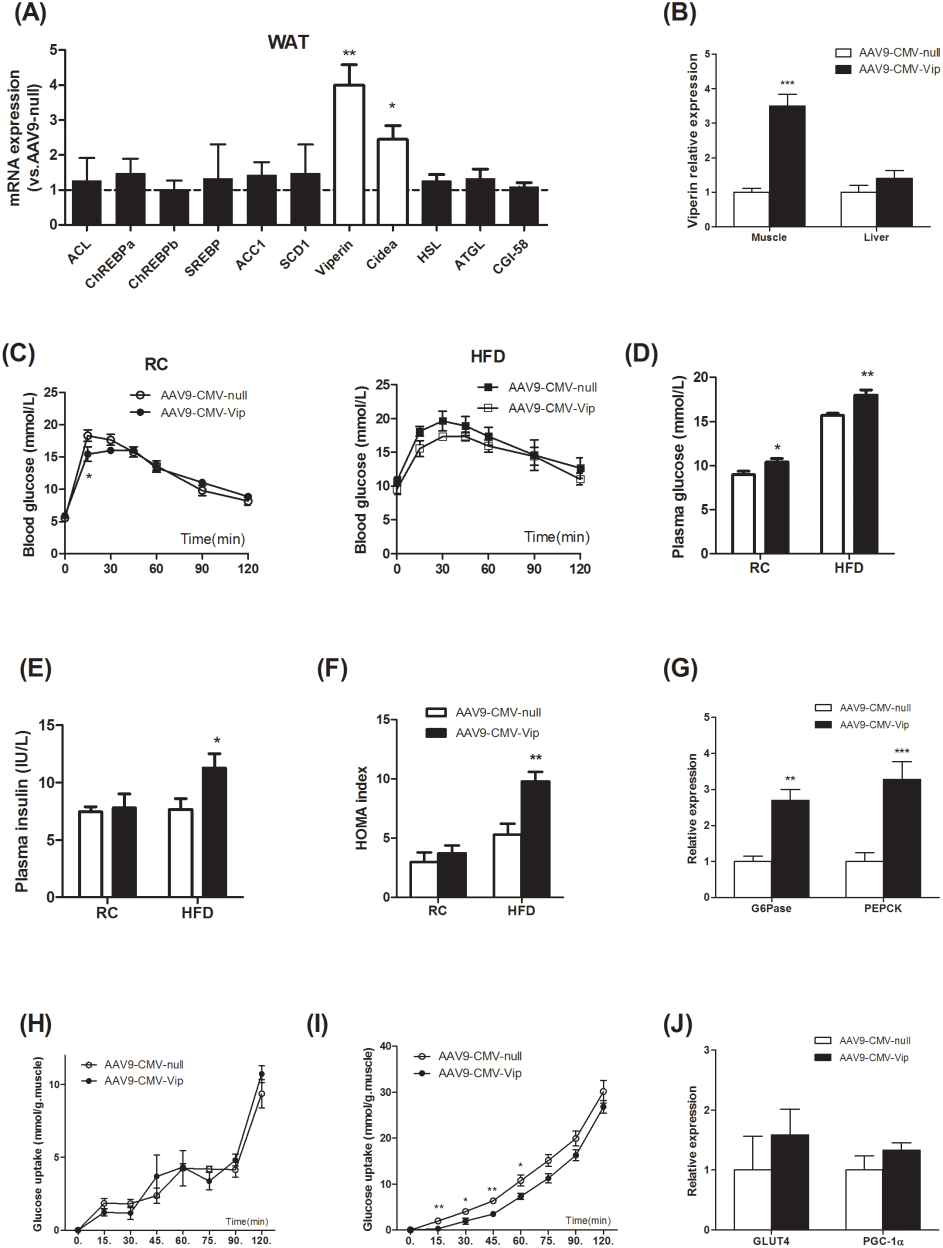
Forced expression of viperin activated gluconeogenesis and reduced glucose uptake in skeletal muscle Mice were intravenously injected with AAV9-CMV-null or AAV9-CMV-Vip only once before sacrifice. Meanwhile, mice were fed HFD for 1 wk. Relative mRNA expression of genes in WAT **(A)**, GAS and liver **(B)** was measured in HFD-fed mice one week after AAV9 injection. Values are normalized to 18S by 2^−ΔΔCt^ method. The data shown are mean ± SE of 6-7 mice per group. ^*^P<0.05, ^**^P<0.01, ^***^P<0.001 versus AAV9-CMV-null. The dot line indicates the values of AAV9-CMV-null in each gene normalized by 2^−ΔΔCt^ method. The data show that AAV9-CMV-Vip targeted WAT and skeletal muscle and increased viperin expression. Blood glucose levels in IPGTT **(C)**, fasting plasma glucose **(D)** and insulin **(E)**, HOMA index **(F)** were measured 1 wk after AAV9 injection. Expression of G6Pase and PEPCK in liver was investigated in HFD-fed mice **(G)**. Values are normalized to 18S by 2^−ΔΔCt^ method. The data shown in Panels C-G are mean ± SE of 6-7 mice. ^*^P<0.05, ^**^P<0.01, ^***^P<0.001 versus AAV9-CMV-null. Intact GAS muscles were extracted from HFD-fed mice and incubated for 15, 30, 45, 60, 75, 90, 120min without **(H)** or with **(I)** insulin 60 mU/mL followed by measurement of 2-deoxy-[^3^H]-glucose uptake. Expression of GLUT4 and PGC-1α in GAS muscle was also investigated in HFD-fed mice **(J)**. Values are normalized to 18S by 2^−ΔΔCt^ method. The data shown in Panels H-J are mean ± SE of 5-6 mice. ^*^P<0.05, ^**^P<0.01, versus AAV9-CMV-null respectively.

We further investigated the effects of acute forced expression of viperin in living animals. Adeno-associated viral (AAV) vectors, serotypes 8 and 9, mediate efficient transduction of white and brown adipose tissue in adult mice. Systemic infusion of AAV vectors encoding target protein enables highly specific and long-term transgene expression in white or brown adipocytes [20]. Here, injection of mice with AAV9 encoding viperin resulted in an increase in the adipose abundance of viperin mRNA and Cidea mRNA, whereas the amounts of mRNAs for lipolysis or lipogenesis were not affected (Figure 3A). In addition, viperin expression was elevated by 3-4 folds in skeletal muscle but not in the liver (Figure 3B).

Blood glucose curve in glucose tolerance test was not affected in mice injected with AAV9-CMV- Vip (Figure 3C). However, forced expression of viperin increased fasting plasma glucose in chow- and HFD-fed mice (Figure 3D), suggesting that gluconeogenesis in fasted state was enhanced. Plasma insulin concentration in fasted state and HOMA index were both elevated by viperin overexpression in HFD-fed mice (Figure 3E, 3F), reflecting the activation of gluconeogenesis in HFD-fed state [21]. Hepatic expressions of G6Pase and PEPCK were upregulated concomitantly by AAV9-CMV-Vip (Figure 3G). Although viperin overexpression could not result in glucose intolerance, our results suggested that the enhanced antiviral potential may induce the overactivity of gluconeogenesis and thus lead to worse metabolic consequences in HFD-fed mice.

To determine whether viperin regulates insulin-stimulated glucose uptake in skeletal muscle *ex vivo*, HFD-fed mice were sacrificed and intact GAS muscles were extracted and incubated for different time with or without insulin (60 mU/mL). During 0-120min incubation, skeletal muscle glucose uptake was irregular in the absence of insulin (Figure 3H). Viperin overexpression had no impact on glucose uptake at any time point (Figure 3H). However, skeletal muscle glucose uptake increased gradually in the presence of insulin. Viperin overexpression repressed glucose uptake during 0~60 min incubation (Figure 3I). These observations suggest that forced expression of viperin further impaired skeletal muscle insulin sensitivity in mice fed HFD, but no significant changes in GLUT4 and PGC-1α expression were found in muscle (Figure 3J).

### Viperin overexpression reduces epinephrine-stimulated lipolysis in adipose tissue

**Figure 4:**
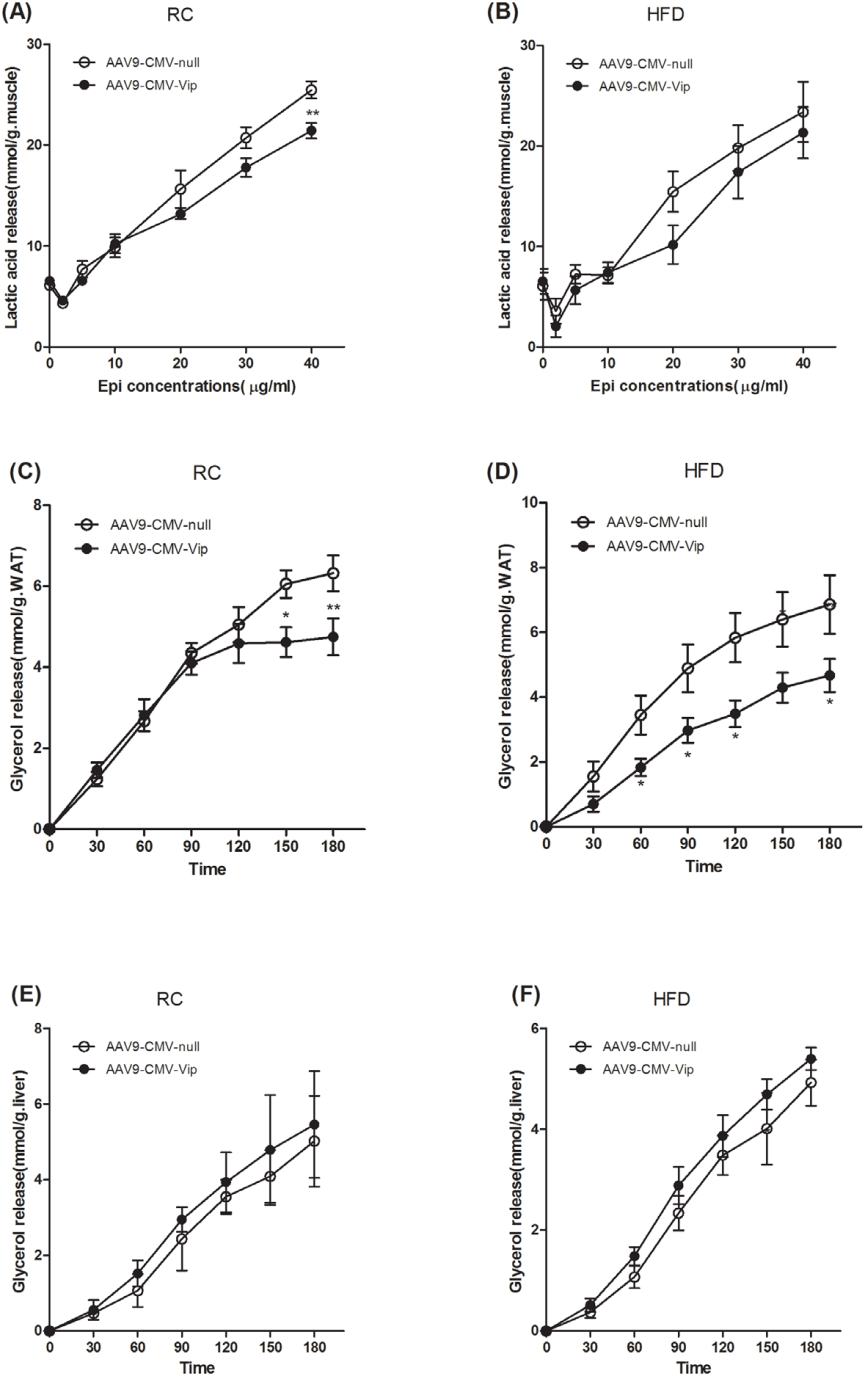
Forced expression of viperin repressed epinephrine-stimulated lipolysis in WAT Mice were intravenously injected with AAV9-CMV-null or AAV9-CMV-Vip only once before sacrifice. Meanwhile, mice were fed HFD for 1 wk. Intact GAS muscle tissues were extracted and incubated with epinephrine (Epi) for 15min at each Epi concentration. Lactic acid was determined in the condition media at the indicated Epi concentrations **(A, B)**. Intact epididymal fat (WAT) was extracted and incubated with epinephrine (5μg/ml for RC, 10μg/ml for HFD) for 180 min **(C, D)**. Liver was extracted and incubated with epinephrine (5μg/ml for RC, 10μg/ml for HFD) for 180 min **(E, F)**. For Panels C-F, glycerol was determined in the condition media at the indicated time points. The data shown in Panels A-F are mean ± SE of 5-6 mice. ^*^P<0.05, ^**^P<0.01, versus AAV9-CMV-null mice at the same time point or Epi concentration.

We have previously shown that catecholamine resistance causes lipid accumulation in WAT by reducing lipolysis, increasing lipogenesis and impeding free fatty acid (FFA) transportation [22]. In this study, we next investigated the effects of acute overexpression of viperin on catecholamine sensitivity *ex vivo*. Intact GAS muscles were extracted and incubated with epinephrine (Epi) for 15min at each Epi concentration. Our data demonstrated that Epi-stimulated lactic acid release depended on the dosage of epinephrine (Figure 4A, 4B). Only at epinephrine 40 μg/ml did viperin overexpression reduce lactic acid release in chow-fed mice (Figure 4A). Viperin overexpression had no impact on Epi-stimulated lactic acid release at any concentration (Figure 4B). Intact epididymal fat (WAT) was also extracted quickly and incubated with epinephrine for 180 min. Our data demonstrated that Epi-stimulated lipolysis (glycerol release) in WAT increased gradually (Figure 4C, 4D). Viperin overexpression repressed lipolysis in chow-fed mice since 150min incubation (Figure 4C), but in HFD-fed mice since 60min incubation (Figure 4D). There was no difference in the rates of glycerol release using the same protocol in liver incubation (Figure 4E, 4F). Our data suggest that forced expression of viperin impaired adipose tissue epinephrine sensitivity, especially in HFD- fed mice.

### Viperin knockdown upregulates lipid mobilization and glucose uptake in HFD-fed mice

**Figure 5:**
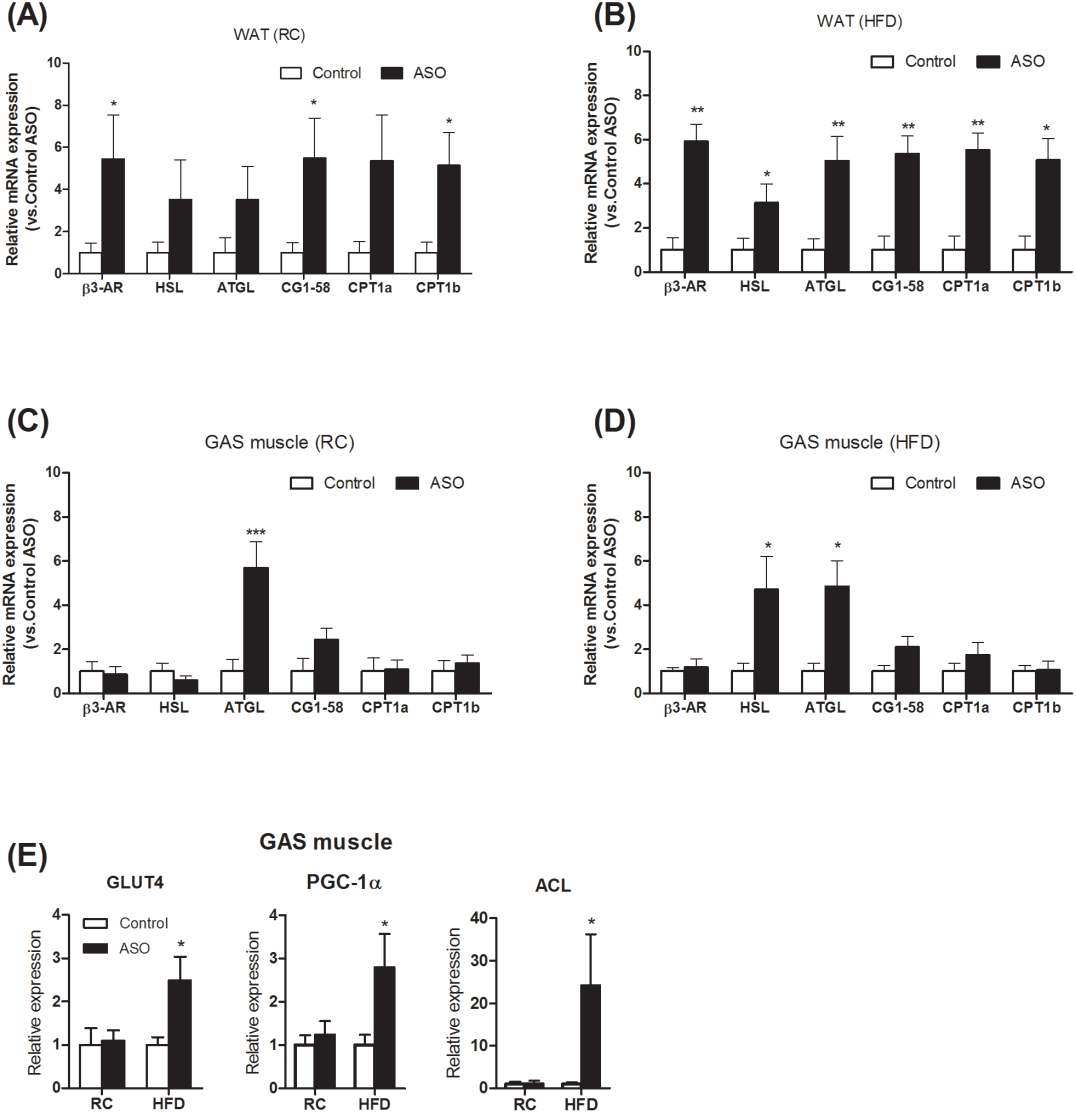
Viperin ASO increased the expression of lipolytic genes in WAT in HFD-fed mice Relative mRNA expression of lipolytic genes was evaluated by qPCR in WAT **(A, B)** and GAS muscle **(C, D)** under regular chow and HFD conditions. **(E)** Change in transcript levels of skeletal muscle genes involved in regulating glucose uptake and utilization. Values are normalized to 18S by 2^−ΔΔCt^ method. The data shown are mean ± SE of 5-6 mice. ^*^P<0.05, ^**^P<0.01, versus control mice at the same diet condition.

**Figure 6:**
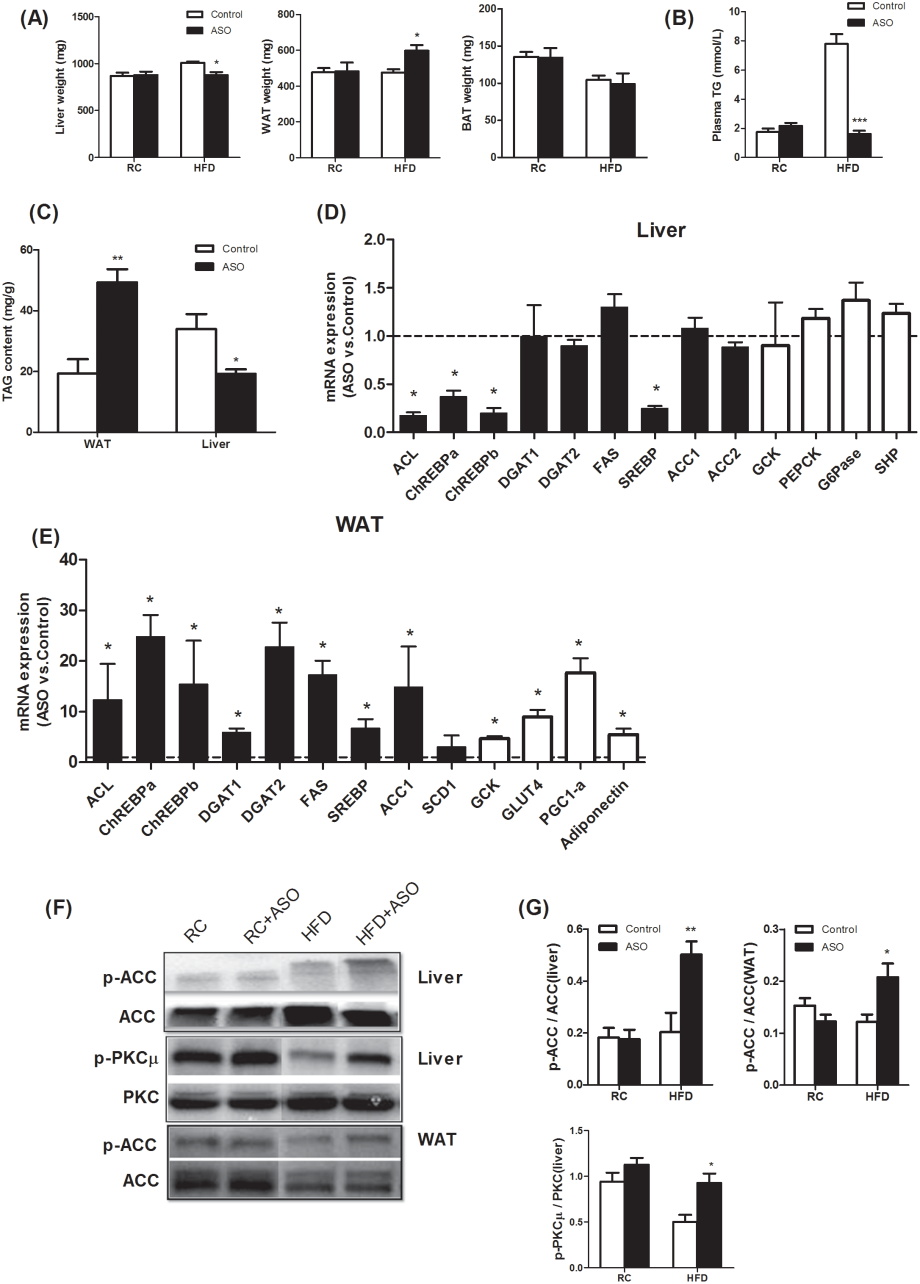
Viperin ASO induced reciprocal regulation of hepatic and adipose lipogenesis in HFD-fed mice **(A)** Tissue weights of liver, WAT, and BAT. Viperin ASO reduced the weight of liver and increased the weight of WAT in HFD-fed mice. **(B)** Plasma TG concentrations in fasted state. **(C)** Adipose and hepatic TAG content in HFD-fed mice. The data shown in Panels A-C are mean ± SE of 5-6 mice. ^*^P<0.05, ^**^P<0.01, ^***^P<0.001, versus control mice at the same diet conditions. **(D)** Real-time PCR analysis of hepatic lipogenesis and gluconeogenesis in HFD-fed mice. **(E)** Real-time PCR analysis of adipose lipogenesis and glucose uptake in HFD-fed mice. Values are normalized to 18S by 2^−ΔΔCt^ method. The data shown are mean ± SE of 5-6 mice. ^*^P<0.05, versus control mice fed HFD. The dot line indicates the values of HFD-fed control mice in each gene normalized by 2^−ΔΔCt^ method. **(F)** Western blot of phospho-ACC and phospho-PKCμ. The blot shown is representative of 2 or 3 others showing the same pattern. **(G)** The quantitative analysis of western blot in Figure 7F. The data shown are mean ± SE of 3-4 mice. ^*^P<0.05, ^**^P<0.01, versus control mice fed HFD.

To understand the molecular pathways by which viperin knockdown improved metabolic control in HFD-fed mice, real-time PCR analysis of lipid mobilization and glucose uptake was performed in WAT and GAS muscle. Our data demonstrated that viperin ASO enhanced the expression of genes for lipolysis and β-oxidation in WAT, especially in HFD-fed mice (Figure 5A, 5B). HSL and ATGL expression were elevated in skeletal muscle (Figure 5C, 5D). Compared with the control mice, the mRNA expression of GLUT4, PGC-1α, and ACL was significantly upregulated not only in skeletal muscle (Figure 5E) and also in WAT (Figure 6E). These genes contribute to glucose uptake, glucose-dependent de novo lipogenesis and mitochondrial function [23–25].

### Viperin knockdown induces reciprocal regulation of adipose and hepatic lipogenesis in the HFD-fed mice

Usually, hepatic steatosis and glucose production are increased and considered to be detrimental in insulin-resistant animals and humans [26–28]. Recently, adipose tissue expansion has been shown to improve the metabolic profile and enhance insulin sensitivity in multiple rodent models [29–31]. In these models, enhanced lipogenesis in WAT did not result in enhanced adipose inflammation and increased risk of metabolic diseases. In our study, viperin ASO protected HFD- fed mice from hepatic steatosis and hyperlipidaemia by reducing liver weight (Figure 6A), plasma TG (Figure 6B) and hepatic lipid content (Figure 6C). Reciprocally, the weight of epididymal fat (WAT, Figure 6A) and lipid content in WAT (Figure 6C) were significantly elevated by viperin ASO. There was no change in the weight of BAT (Figure 6A).

We next analyzed the expression of genes for hepatic lipogenesis and gluconeogenesis in HFD-fed mice. Treating HFD mice with viperin ASO resulted in a significant reduction in the mRNA expression of ACL, ChREBPa, ChREBPb, and SREBP in liver (Figure 6D, black bars). These findings suggest that viperin knockdown, although specially in WAT and skeletal muscle, has the ability to redirect lipid metabolism in whole body. The expression of GCK, PEPCK, G-6-Pase, and SHP was not affected by the knockdown of viperin (Figure 6D, white bars). In HFD-fed mice, viperin ASO increased phosphorylation of ACC and PKCμ (Figure 6F, 6G), representing a therapeutic target for mitigating lipid accumulation, glucose intolerance and maladaptive remodeling [32–34].

For HFD-fed mice, viperin ASO resulted in a remarkable increase in adipose expression profile of lipogenic genes, such as ACL, ChREBPa, ChREBPb, and SREBP (Figure 6E, black bars). Moreover, viperin ASO upregulated the expression of GCK, GLUT4, PGC-1α, and adiponectin, all of which contribute to glucose transport, localization and insulin sensitivity (Figure 6E, white bars). Western blot indicated that viperin ASO in HFD-fed mice enhanced ACC phosphorylation (Figure 6F, 6G), suggesting a potential for AMPK activation in WAT. Together, silencing antiviral protein viperin in HFD-fed mice induced reciprocal regulation of hepatic and adipose lipogenesis, thereby leading to a potential of adipose tissue expansion rather than hepatic steatosis.

### Viperin knockdown exacerbates diet-induced inflammation and macrophage infiltration into adipose tissue

**Figure 7:**
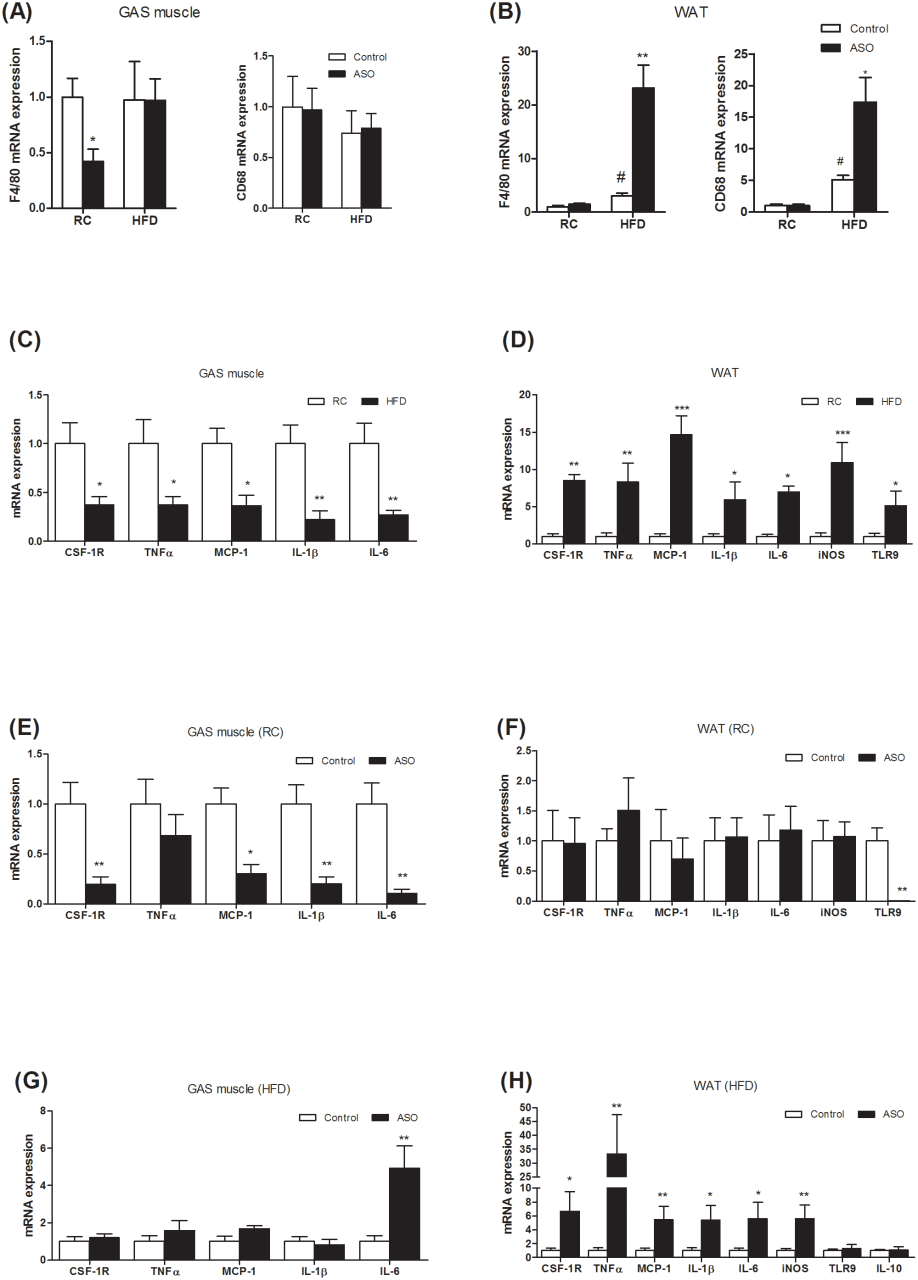
Viperin ASO exacerbated inflammation and macrophage infiltration into adipose tissue, but not into skeletal muscle, in HFD-fed mice **(A, B)** Macrophage marker (CD68, F4/80) mRNA abundance was measured in GAS muscle and WAT. **(C, D)** Effects of HFD on cytokine mRNA expression were also investigated in GAS muscle and WAT. **(E, F)** Effects of viperin ASO on cytokine mRNA expression were evaluated in regular chow-fed mice. **(G, H)** Effects of viperin ASO on cytokine mRNA expression were also evaluated in HFD-fed mice. Values are normalized to 18S by 2^−ΔΔCt^ method. The data shown are mean ± SE of 5-6 mice. ^*^P<0.05, ^**^P<0.01, ^***^P<0.001, versus control mice.

Chronic low-grade inflammation in WAT contributed to the development of obesity-related insulin resistance [1, 2]. Recently, adipose tissue inflammation has been shown to be essential for healthy adipose tissue expansion and remodeling that enables visceral fat depot to effectively filter gut-derived endotoxin [5]. To assess the relevance of adipose tissue inflammation to lipogenesis and glucose homeostasis, we analyzed the expression of genes in the inflammatory response. In the HFD-fed mice, viperin ASO increased the expression of macrophage/monocyte markers F4/80 and CD68 in WAT, but not in skeletal muscle (Figure 7A, 7B). HFD reduced the expression of proinflammatory genes in skeletal muscle (Figure 7C), but increased in WAT (Figure 7D). For regular chow-fed mice, viperin knockdown reduced the expression of CSF-1R, MCP-1, IL-1β and IL-6 in skeletal muscle (Figure 7E), but not in the WAT (Figure 7F). For HFD-fed animals, viperin knockdown further increased the expression of cytokines including TNF-α, IL-1β, IL-6 in the WAT (Figure 7H) and resulted in a 4~5 folds increase in the expression of MCP-1 and iNOS (Figure 7H), which contributes to macrophage infiltration into adipose tissue and mediates adipose tissue inflammation and insulin resistance [35, 36]. IL-6 was also upregulated in skeletal muscle by viperin ASO (Figure 7G). Together, WAT differed so much from skeletal muscle in inflammatory responses when mice were exposed to HFD and viperin ASO. The combination of HFD and viperin ASO developed an abnormal metabolic phenotype, in which improved glucose tolerance was accompanied by enhanced adipose tissue inflammation.

## DISCUSSION

Antiviral treatments have been increasingly used for improving glycemic control and decreasing diabetes risk in patients with HCV infection [37, 38]. Viperin has been identified as an antiviral protein associated with obesity-related diseases by enhancing lipid droplet contents and lipid biosynthesis in humans [39]. We had thought that viperin knockdown would cause deficiency in antiviral activity and thus impair insulin sensitivity in mice. However, we demonstrate here that viperin knockdown counteracts the detrimental effects of HFD feeding on weight, hyperlipidemia, hepatic steatosis and systemic glucose intolerance in mice. Forced expression of viperin represses adipose tissue lipolysis, reduces skeletal muscle glucose uptake, and enhances fasting plasma glucose and insulin concentrations. Unexpectedly, viperin knockdown leads to increased adipose inflammation and macrophage infiltration. Together, viperin silencing may develop an abnormal metabolic phenotype in HFD-challenged mice. We present novel evidence that reducing antiviral potential may promote animal adaptability to HFD challenge.

The first question we need address is the relationship between viperin and insulin resistance. We initially quantified the expression of viperin in different tissues obtained from insulin-resistant mice undergoing HFD and leptin receptor knockout. Viperin expression in adipose tissues was downregulated in the two mouse models, whereas the effects in skeletal muscle and liver were inconsistent. Reduced viperin expression was rescued in the adipose tissue of leptR-/- mice by exercise. We speculate that reduced viperin expression in adipose tissue causes insulin resistance. On the contrary, the following results suggest that viperin knockdown improved diet-induced glucose intolerance and hyperlipidemia. We conclude that reduced viperin expression in adipose tissue may be a consequence not a cause of insulin resistance. This is why viperin ASO could not result in insulin resistance through reducing viperin expression.

Instead, suppressing viperin expression improved glucose homeostasis in HFD-fed mice. This is in line with earlier findings showing that suppressing innate antiviral immunity using cyclosporine A protects against HFD-induced obesity and insulin resistance in mice [40, 41]. Recent data revealed that insulin receptor tyrosine kinase substrate (IRTKS) mediates suppression of antiviral responses. Antiviral action needs to be strictly suppressed by insulin signaling to avoid damage to cell from exacerbated inflammation [42]. Viperin is an IFN-stimulated gene induced by type I (IFN-α, β), II (IFN-γ), and III IFNs or after infection with a range of viruses [43]. However, the induction of IFNs leads to either insulin resistance or increased glucose uptake, depending on the type of IFN. IFN-γ attenuates insulin signaling, lipid storage, and differentiation in adipocytes [44]. IFN-γ deficiency inhibits the inflammatory response of macrophages and subsequently suppresses steatohepatitis induced by HFD [45]. IFNγ-knockout in obesity improved insulin sensitivity and decreased adipocyte size, macrophage infiltration and cytokine expression [46]. Therefore, IFNγ-induced gene expression and inflammatory response promote diet-induced obesity and the metabolic abnormalities in liver and adipocytes. In contrast, IFN-β induces glucose uptake and GLUT4 translocation to cell surface. IFNβ-inducible glucose metabolism diminishes the antiviral response, but increased insulin sensitivity enhances the antiviral potency of IFN-beta [47]. This suggests that the involvement of IFN-β in glucose metabolism impairs its antiviral ability. Previous studies have shown that high-fat diet elicited type I IFN-regulated gene expression and induced metabolic dysfunction, whereas abrogating type I IFN signaling increased susceptibility to HFD and led to worsened steatosis and inflammation [48]. These findings suggest that adipose type I IFN signaling protects against diet-induced metabolic dysfunction, though it mediates inflammatory responses. Another study reported that high-fat diet decreased IFN-γ production in rats [49]. As a target of IFNs family, viperin expression depends on the relative activity of IFN-β and IFN-γ. In this study, it is inconsistent for the relationship of viperin and glucose tolerance in different models. In view of the multiple roles of IFNs in inflammation and glucose metabolism, we conclude that reduced viperin in adipose tissues represents a self-protective consequence to control local inflammation and damage in these insulin-resistant models. However, viperin ASO reduces animal metabolic susceptibility to HFD and improves glucose homeostasis by interfering the induction of IFNs probably.

We next asked if forced expression of viperin by AAV delivery results in glucose intolerance in mice. Indeed, forced expression of viperin significantly increased fasting glucose and insulin levels, but not resulted in changes in systemic glucose homeostasis. Although 1-week HFD increased fasting plasma glucose by 75%, insulin secretion was not elevated until in the presence of viperin overexpression. Skeletal muscle glucose uptake was reduced by viperin overexpression under HFD conditions. These data suggested that forced expression of viperin exacerbated diet- induced metabolic disorders. Usually, viperin expression is induced by IFN to mediate antiviral activity against HCV and HCMV [17, 50]. HCV promotes hepatic gluconeogenesis and results in glucose homeostasis abnormality [51, 52], whereas HCMV increases glucose uptake, glycolysis and fatty acid biosynthesis [53, 54]. Further, HCMV infection activates GLUT4 expression to increase glucose uptake in adipose tissue [55]. In our current model, viperin expression was forced to elevate by AAV delivery, mechanically simulating antiviral signaling activation. Obviously, our results partly replicate the metabolic consequence of HCV infection, but not HCMV. This is in line with recent suggestions that IFN-regulated glycolytic metabolism is important for the acute induction of an antiviral response during viral infection [56, 57]. In this study, all measurements of glucose metabolism were conducted only 1 week after AAV injection, so the increased fasting glucose and insulin may be implicated in the acute induction of the simulated antiviral response. In contrast to acute HCMV infection [18], viperin knockdown activated expression of GLUT4, PGC-1alpha, and ACL in skeletal muscle, suggesting that reducing antiviral potential enhances glucose metabolism to support fatty acid synthesis. After all, viperin is a modulator of lipid metabolism and responsible for phenotypic alterations in adipose, liver and muscle tissues when mouse strains are challenged by high-fat diet [39].

Further, forced expression of viperin dramatically reduced epinephrine-stimulated lipolysis in WAT. In the non-challenged mice (fed chow diet), viperin had limited effect on adipose tissue lipolysis. However, viperin overexpression delayed fat mobilization as soon as mice were exposed to HFD. These interaction effects indicate that enhancing antiviral potential further exacerbates diet-induced dysregulation of lipolysis and lipid metabolism. Previous studies show that HFD inhibited basal, epinephrine-, and forskolin-induced AMPK activation as well as fatty acid oxidation in fat depots [58]. Also, HFD induced steatohepatitis and interferon-stimulated genes in mice [59]. FFA derived from diets increased IFN-driven gene expression that may account for HCV treatment failure [60]. These data suggest that HFD induces the antiviral action of IFNs and therefore mediates progressive inflammation and the consequent insulin resistance [61]. In this study, viperin knockdown increased the expression of genes responsible for epinephrine-induced lipolysis. Our data indicate that silencing antiviral activity promotes adipose tissue lipolysis in HFD-fed mice.

Increasing data demonstrate that enhanced lipogenesis results in ectopic lipid accumulation, insulin resistance, hepatic steatosis and obesity [27, 62–64]. Here, we showed that the knockdown of viperin decreased hepatic lipogenesis but increased expression of lipogenic gene in WAT. Viperin knockdown increased not only activation of hepatic PKCμ, ACC and also adipose ACC and Akt phosphorylation in HFD-fed mice. These findings reveal a role for viperin in promoting reciprocal regulation of hepatic and adipose lipogenesis. In comparison to our findings, human obesity often displays increased hepatic lipogenesis but decreased expression of lipogenic gene in adipose tissue [65]. Similar to our observation, liver X receptors knockout ob/ob mice remain obese but show reduced hepatic steatosis and improved insulin sensitivity. Reduced hepatic lipogenesis is accompanied by reciprocal increases in adipose lipid storage [66]. We speculate that adipose-selective viperin knockdown results in reciprocal regulation of hepatic and adipose lipogenesis, because hepatic viperin expression is so low that liver has no susceptibility to ASO interference. Enhanced phosphorylation of ACC in liver and WAT suggests a potential of AMPK activation, which may contribute to reducing hepatic steatosis and promoting adipocyte lipolysis. It is noteworthy that viperin ASO increased the expression of adiponectin, GLUT4, and PGC-1α, all of which facilitate glucose uptake in adipose tissue. Together, viperin ASO promotes reciprocal regulation of hepatic and adipose lipogenesis, which may account for the improvement of glucose homeostasis.

A large body of literature suggests that infiltrating macrophages recruited to adipocytes stimulate adipose tissue inflammation and impair insulin sensitivity through the production of inflammatory cytokines [1, 67–69]. In human adipose tissues, subcutaneous and visceral/omental levels of complement cascade-derived cytokines (such as C3a, C5a, C5b-9/MAC) and adipocytokines (such as osteocalcin and visfatin) were strongly correlated with individual metabolic status, such as body mass index, adiposity, waist and hip circumference [70, 71]. These findings support that adipose inflammatory factors induce metabolic disorders. In HFD-fed mice, we also observed increases in proinflammatory gene expression associated with macrophages infiltration. This is consistent with data showing that large numbers of CD8(+)T cells infiltrated epididymal adipose tissue in mice fed high-fat diet [72]. Unexpectedly, HFD-fed mice with treatment of viperin ASO exhibited higher proinflammatory response in the WAT, although systemic glucose homeostasis and lipid metabolism were improved in these mice. As discussed, macrophages infiltration into adipose tissue would produce a larger number of proinflammatory cytokines and impair insulin sensitivity and lipid metabolism. In mice with ASO treatment, however, increased inflammatory response in adipocytes was accompanied by an improvement of glucose metabolism. This was no longer a low-grade inflammation, because proinflammatory gene expression in WAT was further increased by 6~30 folds compared to HFD-fed mice. These findings challenge our previous notion that infiltrating macrophages cause adipose tissue inflammation and impair glucose homeostasis. Such an abnormal phenotype has been shown in interleukin-1 receptor I (IL-1RI) and interferon regulatory factor 7 (IRF7) knockout mice. IL-1RI mediates immune signaling to adipose tissue inflammation. IL-1RI knockout enhanced glucose homeostasis without accompanying change in macrophage number in adipose tissue [73]. Furthermore, macrophage-secreted HIF-2α counteracts proinflammatory responses to relieve obesity-induced insulin resistance in adipose tissue [74]. Thus, infiltrating macrophages play a dual role in inducing adipose tissue inflammation. IRF7 is also a mediator of type I interferon-dependent immune responses. Similar to our findings, IRF7 knockout improved glucose homeostasis and insulin sensitivity and ameliorated diet-induced hepatic steatosis [61]. With these findings, our data on viperin knockdown support the notion that reducing local immune responses alleviates insulin resistance.

Another question we need address is how reducing antiviral potential with ASO further enhances adipose tissue inflammation. There is a possibility that the antiviral response of viperin is initially provoked by virus infections and inflammation, and ultimately alleviates inflammation so as to protect cell from viral infections. The ultimate consequence of antiviral action is to suppress inflammation, so the deficiency in antiviral response results in enhanced inflammation. Studies have shown that mice lacking viperin had higher viremia and severe joint inflammation compared with wild-type mice [75]. Reducing antiviral potential may impair cell capability to eradicate virus and alleviate inflammation. Therefore, viperin ASO further enhanced HFD-induced adipose tissue inflammation. Although knockdown of viperin improves glucose homeostasis in HFD-fed mice, the risk for reduced antiviral potential remains to be elucidated.

**Figure 8:**
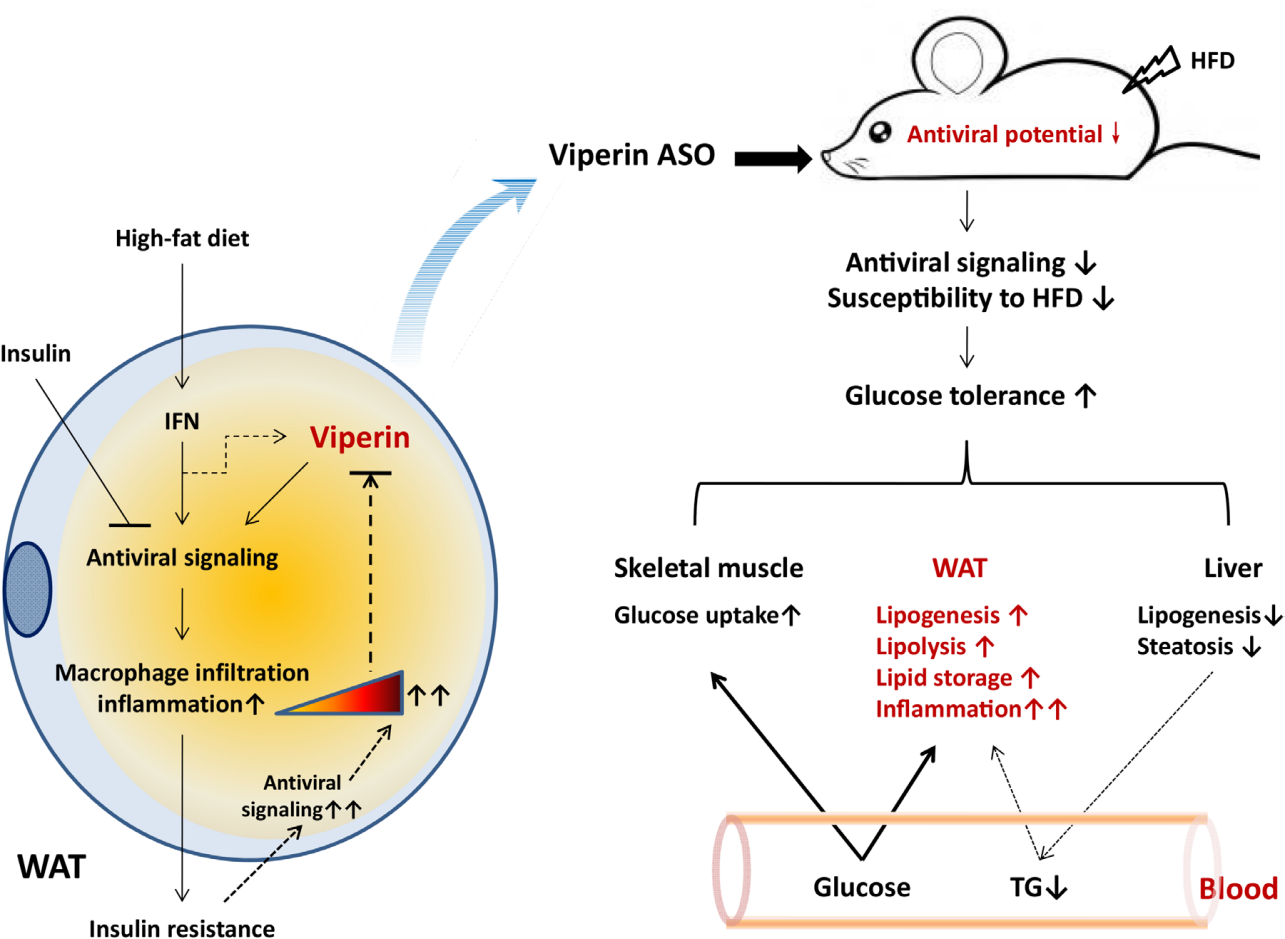
Proposed model for the role of viperin in regulating glucose homeostasis Left panel, exposure to high-fat diet activates the antiviral signaling of IFN in adipocytes, thereby inducing macrophages infiltration into adipose tissue and inflammation. Insulin signaling can moderately suppress the antiviral response and thus avoid damage to adipocytes from excessive inflammation. Therefore, diet-induced insulin resistance may further increase the antiviral response and result in excessive inflammation in adipose tissue. To some extent, there may be a self-protective feedback mediated by viperin downregualtion to attenuate antiviral response moderately in adipocytes. **Right panel**, when mice were exposed to HFD and antiviral signaling would be provoked in WAT, viperin knockdown could reduce adipose antiviral potential and susceptibility to HFD. Viperin ASO protected against diet-induced glucose intolerance through the reciprocal regulation of adipose and hepatic lipogenesis and the increased glucose uptake in skeletal muscle and WAT. There may be an unknown mechanism to induce hepatic lipid into adipocytes through the cycle of plasma TG. On the other hand, adipose tissue inflammation was further enhanced by viperin ASO due to reduced antiviral activity in adipocytes, even if glucose homeostasis in whole body was improved.

In summary, our results suggest that the antiviral protein viperin is a potential target to improve glucose homeostasis and hepatic lipid metabolism. Exposure to HFD provokes antiviral signaling and inflammatory response, but the consequence of insulin resistance attenuates insulin-mediated suppression of antiviral responses. Reduced viperin in adipose tissues represents a self-protective response to HFD. Our findings show that silencing viperin improves diet-induced metabolic disorders, such as glucose intolerance and hyperlipidemia, but not adipose tissue inflammation. These effects appear to be consequences of reciprocal regulation of adipose and hepatic lipid metabolism (summarized in Figure 8). We suggest that the beneficial effects of silencing viperin on glucose homeostasis and hepatic protection need rely on antiviral impairment of adipose tissue. Further studies are needed to investigate how viperin is induced by IFNs in different tissues and assess the risk of virus infection in viperin-silencing animals.

## MATERIALS AND METHODS

### Mice

C57BL/6 mice were purchased from Shanghai SLAC laboratory Animal Co., Ltd (SLAC). LeptR-/- mice, on a C57BL/6 background, were purchased from Model Animal Research Center of Nanjing University. All animal procedures were conducted under protocols approved by the animal experimentation committee of East China Normal University. Body weight of the animals was recorded weekly. Grouped male mice (4~5 weeks of age at the start of experiment) were used in the diet-intervention experiment. Mice were given regular chow (RC) or high-fat diet (HFD), containing 4 or 45% kcal derived from fat, respectively. The care and use of the animals were in accordance to China guidelines for care of experimental animals. The general health of the animals was monitored daily by the professionals. The study protocol caused minor suffering; however, animals were deeply anesthetized with 5% Isoflurane at the day of sacrifice.

### ASO treatment and recombinant adeno-associated virus (AAV)

Mice received intraperitoneal injections of ASO against Viperin or a scrambled control ASO 25 mg/kg body weight twice a week for 4 weeks. ASOs used for i.p. injections were resuspended in sterile 0.9% saline. The most active compounds were screened *in vivo* to select the ASO used in this study. The ASO sequences are as follows: control ASO 5′-CCTTCCCTGAAGGTTCCTCC-3′ and viperin ASO 5′-ATCCC TGCACCACCTCCTCA-3′ (Sangon Biotech (Shanghai) Co., Ltd.). For the ectopic expression of viperin, single-stranded AAV (ssAAV) vectors were produced by triple transfection of HEK293T cells and purified by a CsCl-based gradient method. A noncoding plasmid carrying the CMV promoter was used to produce null particles (AAV9-CMV-null) as described previously [20]. AAV9 vectors encoding viperin under the control of the ubiquitous CMV promoter (AAV9-CMV-Vip) or an equal dose of the AAV9-CMV-null vector were delivered to eight-week-old C57BL/6 mice by the tail vein (4.69×10^10^/mouse).

### Intraperitoneal glucose and insulin tolerance test (GTT, ITT)

Mice were deprived of food for 16 h and then subjected to glucose or insulin tolerance test after 4 weeks of ASO treatment or 7 days after AAV injection. Blood was collected from a small incision in the tip of the tail (time 0) and then 15, 30, 45, 60, 90 and 120 min after an i.p. injection of glucose (1g/kg body weight) or insulin (0.75 U/kg body weight). Blood glucose levels were measured with a blood glucometer (Accu-Check^®^ Active, Roche).

### Skeletal muscle glucose uptake and epinephrine tolerance test *ex vivo*

Intactgastrocnemius muscle and epididymal fat (WAT) from mice were preincubated for 2 h at 30°C in oxygenated (95% O2 and 5% CO2) Krebs-Ringer bicarbonate HEPES buffer (KRBH) containing (mM): 129 NaCl, 5 NaHCO_3_, 4.8 KCl, 1.2 KH_2_PO_4_, 1.2 MgSO_4_, 2.5 CaCl_2_, 2.8 glucose, 10 HEPES, and 0.1% BSA at pH 7.4. For *ex vivo* glucose uptake assay, intact muscles were incubated in culture medium with or without insulin (60 mU/mL) in an independent test for 15, 30, 45, 60, 75, 90, or 120 min. 2-deoxy-[^3^H]-glucose uptake was measured after incubation. For *ex vivo* analysis of epinephrine- stimulated lactic acid release, intact muscles were incubated for 15 min with epinephrine 2, 5, 10, 20, 30, or 40μg/mL, and then lactic acid content in culture medium was measured after incubation. For *ex vivo* analysis of epinephrine-stimulated glycerol release, WAT and liver were respectively incubated for 180 min with epinephrine. Glycerol contents in culture medium were measured per 30 min. Lactic acid and glycerol contents were measured using spectrophotometer following the manufacturers’ protocols (Nanjing Jianchen Biotech).

### Plasma parameters

Plasma concentrations of insulin were measured by ELISA. Plasma glucose, triglycerides (TG, TAG), free fatty acid (NEFA), and cholesterol were determined with enzymatic colorimetric assay following the manufacturers’ protocols (Nanjing Jianchen Biotech).

### Tissue lipid measurement

Tissue TAG was extracted using the method described previously [76]. Pieces of tissues were placed in preweighed glass vials and weighed. Tissues were homogenized in ice cold 2:1 chloroform: methanol, and lipids were extracted with shaking at room temperature for 3-4 h. Sulfuric acid was added to ~100 mM, and samples were vortexed and then centrifuged to achieve phase separation. The organic phase was then transferred to another preweighed vial (the lipid vial). The extraction medium in the lipid vial was evaporated, and TAG was measured using a Triglyceride Reagent Kit following the manufacturers’ protocols (Nanjing Jianchen Biotech).

### Real-time PCR

Total RNA was isolated from tissues using TRIzol (Invitrogen), according to the manufacturer's instructions. RNA was reverse-transcribed (cDNA Synthesis Kit, TOYOBO, Osaka, Japan). cDNA was amplified by real-time PCR in a total reaction volume of 20μl using SYBR Green Realtime PCR Master Mix (QPK-201; TOYOBO, Osaka, Japan). Real-time PCR reactions were cycled in StepOne™ Real-Time PCR System (Applied Biosystems). Target gene expression was normalized to housekeeping gene 18S or GAPDH and expressed as 2^-ΔΔct^ relative to the control group, respectively.

### Western blot

For frozen tissue, lysates were prepared using a RIPA lysis buffer (50mM Tris (pH7.4), 150mM NaCl, 2mM EDTA, 1% Nonidet P-40, 50mM NaF, 0.1% SDS and 0.5% sodium deoxycholate with PhosStop Phosphatase-Inhibitor Cocktail tablet and protease-inhibitor cocktail tablet (Roche). The homogenate was centrifuged at 4°C for 10 min at 14,000g and the supernatant was used for western blot. The protein content of the supernatant was quantified using bicinchoninic acid reagents and BSA standards. Equal amounts of protein were separated using a polyacrylamide SDS-PAGE gel. After SDS-PAGE, proteins were transferred to PVDF membrane. The membrane was blocked for 1 h at room temperature followed by incubation overnight at 4°C with viperin antibody (Abcam), phospho-PKCμ, phospho-ACC, phospho-Akt, or GAPDH antibodies (Cell Signaling). After overnight incubation, the blots were incubated with HRP- conjugated secondary antibodies at a dilution of 1:5,000 for 1 h at room temperature. Bands were visualized by ECL plus (Thermo Scientific) according to the manufacturer's instructions and quantified using Image lab software.

### Statistical analysis

Data are shown as Mean ± SEM. Differences between groups were analysed statistically using Student's t test, and differences among the four groups were analysed with analysis of variance followed by post-hoc Bonferroni tests in Graphpad Prism 5.0. P-value < 0.05 was considered significant.
